# Caregiver response types and children language outcomes in preschoolers with and without hearing loss in Aotearoa New Zealand

**DOI:** 10.1080/03036758.2024.2356183

**Published:** 2024-06-05

**Authors:** Nuzhat Sultana, Aleah S. Brock, Suzanne C. Purdy

**Affiliations:** a School of Psychology, University of Auckland, Auckland, New Zealand; b Department of Counseling, Higher Education, and Speech-Language Pathology, University of West Georgia, Carrollton, GA, USA; c Eisdell Moore Centre for Hearing and Balance Research, Auckland, New Zealand

**Keywords:** Language outcome, language input, response types, coaching for caregivers, early intervention

## Abstract

Recent advances in audiological early intervention and hearing technologies have significantly improved access to spoken language for children with hearing loss (CwHL), but many CwHL require additional support to match the language development of their peers with normal hearing (PwNH). Programmes such as It Takes Two to Talk®, the Hanen Program® and Talking Matters focus on supporting parents to enhance children’s language development in natural environments. Analysis of response types has become a significant trend, facilitated by technological developments like Language ENvironment Analysis (LENA®), which provides uninterrupted recordings and automated calculations of adult–child interactions. This research examined three child language measures, child vocalisation counts, total number of words, and mean length of utterance, comparing CwHL and PwNH. Transcribed excerpts of LENA recordings were coded to determine caregivers’ use of ‘high-level’ responses in exchanges with their children and were correlated with child language outcomes. The results confirm the crucial role of caregiver response types in enhancing child language outcomes and exemplify the bidirectional relationship of caregiver-child interactions. The findings add to the literature to suggest that families and educators would benefit from guidance and coaching to acquire and apply high-level response types during natural spoken language interactions with CwHL.

## Introduction

We are in an exciting era for those committed to supporting the development of spoken language skills in young children with hearing loss (CwHL) (Nicholas and Geers 2006; Fulcher et al. 2012; Moeller and Tomblin 2015; Davidson et al. 2021). Language outcomes have been significantly enhanced by early identification via newborn hearing screening and early access to intervention services (Fulcher et al. 2012). Timely fitting of hearing aids or cochlear implants plays a crucial role, providing vital auditory access essential for spoken language development, fostering improved speech perception and language skills (Ching et al. 2013). Engagement in specialised early intervention programmes tailored for CwHL has also significantly contributed to enhanced language abilities (Wright et al. 2021).

Despite great progress in identifying hearing loss early and providing quick access to support and advanced hearing technology for CwHL (Wake et al. 2005; Tomblin et al. 2014; Stika et al. 2015), some CwHL still struggle to develop spoken language skills in line with their typically developing peers with normal hearing (PwNH) (Aragon and Yoshinaga-Itano 2012; Välimaa et al. 2022). Understanding differences in spoken language outcomes between CwHL and their PwNH can help us identify the additional supports needed to close these gaps.

### Quantity-based language outcomes

Mean Length of Utterance (MLU) is an indicator used to assess the complexity of a child’s utterances. Although MLU is a simple measure, it is likely that children with a higher MLU have more advanced language (Geers et al. 2003; Nittrouer 2010). A few studies have compared spoken language outcomes between CwHL and PwNH using key quantitative measures such as total number of child vocalisations (CVC), child total number of child words (TNW) produced, and child MLU (Nittrouer 2010; Ambrose et al. 2014). Ambrose et al. (2014) observed that 2-to-3-year-old children with mild-to-moderate hearing loss had reduced number of vocalisations and words produced, and shorter MLU compared to their PwNH, despite the hearing loss being identified early. The impact of early versus late identification was examined as a contributing factor for language delay by Nittrouer (2010) in her longitudinal study of children with hearing aids or cochlear implants. Among those not using sign language, early-identified children had MLU values of 2.40, 2.70, and 3.02 morphemes at 36, 42, and 48 months, while late-identified children had lower values of 1.99, 2.48, and 2.90 morphemes, respectively. Geers et al. (2003) studied 8- to 9-year-old 181 children with cochlear implants and found that their MLUs were also significantly lower than typically developing PwNH.

### Quality-based language input: ‘response types’

Caregivers are pivotal as active agents in their child’s development and language learning skills. Although quantity of language input is crucial for development of language skills, researchers have emphasised that quality of language input also significantly impacts language acquisition and overall language skills of CwHL, underlining the importance of enriched and varied language environments for supporting linguistic proficiency (Moeller 2000; DesJardin and Eisenberg 2007; Fulcher et al. 2012; Rowe 2012; Cruz et al. 2013; Ambrose et al. 2015).

A comparison of quality of language input between CwHL and PwNH based on caregiver response types has been the focus of several studies exploring language development in CwHL (Eyberg et al. 2005; Cruz et al. 2013; DesJardin et al. 2014). ‘Response type’ refers to the various ways caregivers react or respond during caregiver-child routine linguistic interactions, in conversation or other communication situations. Toddlers whose interactions with their caregivers include high-level language facilitative techniques (i.e. talk in which the caregiver adheres to the child’s focus) have steeper linguistic trajectories relative to children who experience lower-level interactive talk (Landry et al. 2001). The terminology in the literature does tend to refer to these things using many different terms, including facilitative language techniques and responsive interactions.

High-level linguistic responses which foster a linguistically rich environment for children’s language development include expansions (e.g. completing the child’s verbalisation accurately using a more grammatical and complete language model with the addition of one or more words, without adding new information), ‘wh’ questions (e.g. using a ‘wh’ question and a phrase or sentence as a simple justification for the child to give an answer using more than two words), recast question (e.g. rephrasing the child’s vocalisation in question form), reasons (e.g. providing a simple explanation about context to support effective communication skills), and more (Eyberg et al. 2005; Cruz et al. 2013; DesJardin et al. 2014). DesJardin et al. (2014) found that caregivers of children with normal hearing utilised high-level responses (‘wh’ question, expansions, recast) with their children who had better language skills, and these were significantly and positively related to the children’s verbal language abilities.

Studies investigating caregiver interactions with CwHL compared to PwNH have revealed distinct patterns in the use of ‘wh’ questions, expansions and directives. These differences in caregiver response types may potentially impact language acquisition of CwHL compared to their PwNH. Moeller (2000) and DesJardin and Eisenberg (2007) found that caregivers interacting with CwHL posed fewer and less complex ‘wh’ questions and offered fewer expansions compared to caregivers interacting with PwNH. Similarly, DesJardin et al. (2014) found the majority of parents of CwHL continued to use lower-level interactions (such as close-ended questions) with their children who were at higher levels of language development. Since the continued use of lower-level strategies with children at more advanced levels of linguistic development may hinder their continued language growth (Cruz et al. 2013), the authors postulated that caregivers of CwHL may benefit from intervention directed at helping them transition to using higher-level interaction styles (DesJardin et al. 2014). There is also evidence for differences in caregivers’ use of more directive and corrective response types between CwHL and their PwNH (Cruz et al. 2013; Ambrose et al. 2015).

In accordance with terminology that is prevalent in the current literature, we use the term ‘quality’ to describe high-level interactions between caregivers and their children. However, it is important to note that the use of the word ‘quality’ in reference to caregiver linguistic input has been challenged as potentially ignoring cultural differences (Kuchirko 2019). Indeed, differences in caregiver response types have been reported across different contexts and different communities (e.g. Camaioni et al. 1998; Girolametto et al. 2002; Tulviste 2003). For example, Camaioni et al. found that urban Italian mothers used less directive, and more ‘child-centred’ response types than mothers living rurally. They hypothesised that these differences reflected different life environments and childrearing practices in the two sites. This highlights the importance of looking at the language environment of CwHL across different contexts.

There is evidence that the use of specific high-level response types (wh-questions, expansion, explanation, recast-questions) is associated with higher levels of caregiver education and socioeconomic status (SES) (Sultana et al. 2020), highlighting the need to consider these demographic factors when comparing populations.

### Association between caregiver response types and language outcomes

Previous studies have described how different response types relate to children’s language acquisition in early childhood (Girolametto and Weitzman 2002; Tulviste 2003; Eyberg et al. 2005; DesJardin and Eisenberg 2007; Cruz et al. 2013; DesJardin et al. 2014). DesJardin et al. (2014) found that low-level response types (pointing and labelling) were associated with lower language comprehension scores in CwHL. In a longitudinal study of 97 CwHL aged less than 5 years, Cruz et al. (2013) separated response types into two categories (high- vs. low-level), and found that high-level response types (e.g. parallel talk, wh-questions, expansion, explanation, and recast-questions) were significantly associated with better receptive and expressive language outcomes, in contrast with low-level response types (e.g. linguistic mapping, comments, imitation, labelling, directives, and close-ended questions).

Some studies have examined response types that optimise language development across different age groups. Generally, the use of response types such as imitation, labelling, pointing, commenting on the child’s actions, and directing commands to the child tend to facilitate language learning skills in children with normal hearing during early language emergence stages (0–2 years) (Eyberg et al. 2005). A longitudinal study by Rowe (2012) highlighted the importance of expansions and narrations for stronger vocabulary development in children with normal hearing aged 12–48 months. When children reach 2–6 years, open-ended/wh-questions, expansion, and comments can promote the use of more advanced grammatical sentence structure and phrases.

### The current study

The literature clearly shows that the quantity of language input and different types of responses used by caregivers interacting verbally with their children are both pivotal for language development. In previous studies, language facilitative strategies have often been classified as either high- or low-level (DesJardin and Eisenberg 2007; Cruz et al. 2013). However, these studies primarily focused on the association between language strategies/response types and language outcomes, typically using standardised assessments for receptive and expressive language skills in CwHL. To build upon these findings and adopt a more targeted approach, we chose to exclusively investigate high-level response types including language outcomes (CVC, TNW, MLU) using LENA recordings in our current study. This refined focus enables clearer interpretations of the connection between specific response types and quantitative analyses of language outcomes. The current study aims to answer two research questions:

1. How do child language outcomes (CVC, TNW, MLU) and caregiver high-level response types (specifically, Wh-question (Wh-Q), Expansion (EX), Reason (RS), Recast-question (RC-Q)) differ between CwHL and PwNH during natural caregiver-child interactions?
2. Are differences in quantity (CVC, TNW, MLU) of language outcomes associated with exposure to specific caregiver response types in CwHL and PwNH?

Based on previous research, we hypothesised that quantity of language outcomes would differ between groups and greater quantity of language outcomes would be significantly associated with more high-level caregiver response types.

## Method

Ethical approval was obtained from the Human Research Ethics Committee via the Human Participants Ethics Committee of the University of Auckland, and the Programme Research and Development Committee of the Hearing House (Auckland). Approved information and consent forms for the families were distributed by the participating centres (The Hearing House, Listening and Language Clinic, The University of Auckland, ECCs).

### Participants

A convenience sample of 14 CwHL was recruited from the Hearing House (one of the biggest non-profit organisations for people with hearing loss in New Zealand), the Listening and Language Clinic (the University of Auckland), and Preschools/Early Childcare Centres (ECCs) from Auckland, New Zealand. The recruitment process specified the inclusion criteria (Table 1). All CwHL used either hearing aids or cochlear implants and they were engaged in auditory-verbal therapy (AVT) twice in a month. AVT is an intervention approach that emphasises development of spoken language through listening during caregiver/parent–child interactions in daily routines involving the child and family (Estabrooks et al. 2016). Other communication options such as sign language, gesture and lip reading were not considered for the current study due to the use of LENA technology which can only record caregiver-child verbal interactions.

**Table 1.** Inclusion criteria and individual characteristics of children with hearing loss (CwHL) and typically developing peers with normal hearing (PwNH).

Characteristics	CwHL = 14	PwNH = 20
Participant inclusion criteria	Children’s age = 2–5 yrs Not diagnosed with developmental or learning/intellectual delay/disability English = main language at home Participated in newborn hearing screening Hearing aid fitted by the age of 6 months Where appropriate, cochlear implant received before 2 years of age Engaged in auditory verbal therapy (45–60 min/ each session, twice in a month) Did not use any local and international sign language	Children’s age = 2–5 yrs Not diagnosed with developmental or learning/intellectual delay/disability English = main language at home Normal hearing based on newborn hearing screening pass result and lack of caregiver concern
Sex	Male = 6, Female = 8	Male = 9, Female = 11
Age HL identified	1–6 months (*M* = 3.43, SD = 1.22)	N/A
Level of HL	Bilateral moderate to profound HL	N/A
Type of device	Bilateral hearing aids or cochlear implants	N/A
Age first received amplification	3–14 months (*M* = 6.82, SD = 3.21)	N/A

Notes: Amplified hearing age is based on when the children were first amplified. All devices were optimised for audibility of speech using individual real ear measurements and Speech Mapping (hearing aids) and/or Aided hearing thresholds (cochlear implants). HL = hearing loss.

All CwHL were consistent hearing technology users (all waking hours/per day), based on data logging and reports from their Hearing House audiologist during regular 2-monthly reviews. Detailed information regarding protocols for hearing aid/cochlear implant fitting criteria are provided in Sultana et al. (2024). Audiological information about the CwHL is provided in Table 1. All CwHL were enrolled in ECCs three-days per/week and received individual and group speech language therapy/AVT twice in a month (Table 2).

**Table 2.** Descriptive data for family and child demographics of children with hearing loss (CwHL) and typically developing peers with normal hearing (PwNH).

Family Factors	CwHL (*N* = 14)	PwNH (*N *= 20)
	*M* (SD), Range	*M* (SD), Range
Mothers’ level of education	7.29 (0.73), 6–8	6.80 (2.57), 3–10
Fathers’ level of education	7.14 (0.77), 6–9	6.30 (2.27), 3–10
Socioeconomic level of deprivation (NZDep2013 Index of Deprivation)	3.43 (1.34), 1–5	5.30 (2.87), 1–10
Number of adults in the household (including parents)	2.71 (0.83), 2–4	3.35 (1.31), 2–6
Amount of reported maternal interaction on the weekend (hours)	10.86 (1.88), 6–12	11.1 (2.10), 6–13
Amount of reported maternal interaction on the weekday (hours)	5.93 (1.49), 4–10	4.90 (1.64), 2–9
**Child Factors**		
Age at recording (months)	36.21 (12.44), 25–57	39.90 (12.63), 24–58
Number of siblings	2.57 (0.85), 1–4	1.10 (1.60), 1–4
Birth order	2.36 (0.84), 1–4	1.06 (0.88), 1–4

Notes: Parental education ranged from 3 to 10 levels (3 = certificate to work in specified field/area, 4 = certificate to work or study in broader and specified field/area, 5 = a certificate for technical knowledge and skills within a specific field and study, 6 = a certificate for theoretical and technical knowledge and skills within a specific field and study, 7 = bachelor’s, 8 = bachelor’s honours, 9 = master’s, 10 = doctoral). Socioeconomic level of deprivation falls between the number 1 and 10 [1 = least deprived areas (high socioeconomic status), and 10 = most deprived areas (low socioeconomic status)].

A convenience sample of 20 PwNH was recruited from ECCs. The first author contacted managers of local ECCs via email and phone calls and visited the ECCs to discuss the objectives and other matters related to the study to obtain their agreement to recruit participants. All PwNH were attending ECCs five days per week.

Children in both groups (CwHL, PwNH) were recruited from monolingual English-speaking families with both parents having normal hearing. Ethnicity was predominantly New Zealand European for both groups (86% of CwHL, and 60% of PwNH), 7% of children in both groups were Asian ethnicity, and 40% of PwNH were Māori. There were no Māori participants in the CwHL group, a limitation of the study.

### Materials

The Language ENvironment Analysis (LENA) system was used to record and analyse natural audio recordings. LENA utilises a small device (often referred to as a ‘talk pedometer’) worn on the child’s body to record the child's natural language environment for up to 16 h continuously. Data are uploaded to the system, which uses algorithms to provide an estimate of the number of child vocalisations (CVC), adult words (AWC), and conversational turns (CTC) recorded during the duration of the sample (Ford et al. 2009). Specific details of these recordings are available elsewhere (Sultana et al. 2020). CVCs account for the number of speech-related sounds (e.g. babbling, words) produced by the child (not including vegetative or crying sounds). Vocalisations with more than 300 ms of separation are coded as distinct vocalisations. Total CVC for each child per hour/per day was calculated by the LENA software.

The LENA software currently automates the estimation of CVC, however, other quantitative measures such as TNW and MLU are not calculated, and the software cannot determine caregiver response types such as Wh-Q, EX, RS, and RC-Q (Table 3). These were determined using manual transcription and coding of selected segments of audio samples from the LENA recordings.

**Table 3.** Caregiver response types with descriptions and examples.

Response Types	Description	Examples
‘WH’ Question (Wh-Q)	Use a ‘w*h*’ question and a phrase or sentence as a simple justification for the child to give an answer using more than two words.	The parent asks ‘why are you interested in listening to this story’?
Recast Question (RC-Q)	The parent rephrases the child’s vocalisation as a question.	The child says, ‘Anna went … ’ and the mother says, ‘Where did Anna go’?
Expansion (EX)	The parent repeats the child’s verbalisation and completes it accurately using a more grammatical and complete language model with the addition of one or more words, without adding new information.	The child says, ‘Doggie goes … ’ and the parent says, ‘The dog is going’. Or the child says, ‘Baby cry … ’ and the parent says, ‘The baby is crying’ etc.
Reason (RS)	The parent provides child with a simple explanation about why they need to carry out a talk.	The parent says, ‘You should wash your hands because they are dirty’.

### Procedure

After obtaining consent, the first author contacted families to discuss the use of the LENA audio recorders in more detail and liaise with families to deliver and return the LENA recorders. Children wore the LENA recorder in a vest for all waking hours during usual activities (e.g. shopping, staying home), except swimming/bath time. Families were instructed not to record on days with special occasions (e.g. birthday parties, family gatherings) and were asked to behave naturally and interact with their children as usual. The primary researcher was available by phone or e-mail during the recording periods to answer questions. Families were informed that if they felt uncomfortable with the recording due to an unusual day, they could stop recording or withdraw their participation at any time during the data collection process; no parents requested this. Parents were informed that child vocalisation would be calculated automatically, and some parts of recording segments would be transcribed to calculate TNW and MLU and coded to examine caregiver response types. Caregivers were not told that the study would examine specific types of responses within specific times of the day, in order not to bias caregivers.

Only data from weekend days were included to evaluate differences in quantitative measures of language outcomes (CVC, TNW, MLU) and caregiver response types, due to children attending their preschool/ECC on weekdays. Although there was a difference in ECC attendance between groups, there was no significant difference between CwHL and PwNH in the number of hours that caregivers reported spending with their child during weekday or weekend days. During weekend days family members were likely to be at home and engaged in routine child centred activities, e.g. waking up, eating meals, playing together, and reading books, etc, providing a robust indication of typical caregiver-child verbal interactions in a natural environment (Table 2)

#### Selection of audio-recordings for analysis

##### CVC

LENA recording durations over the two weekend days varied slightly in terms of total recorded minutes (Weekend Day 1 = 593–877, Weekend Day 2 = 569–881) for both groups (CwHL and PwNH), but these differences were not significant (Weekend Day 1: *Mdn* = 779.50, *IQR* = 190.75 vs. Weekend Day 2: *Mdn* = 789.50, *IQR* = 110.50, *z* = −0.45, *p* = .655). Slight differences in terms of minutes in duration could lead to an increase or decrease in CVC. To correct for these variations in recording time across families and days, CVC/min values were calculated for each participant by dividing the observed daily values by the number of total recorded minutes and converting the results to CVC/hr, as recommended by VanDam et al. (2012).

##### TNW, MLU and caregiver response types

To identify TWN and MLU and caregiver response types (WH*-*Q, EX, RS and RC-Q), 20 min of LENA audio recordings for each participant (i.e. two per day for two weekend days, resulting in four 5-min recordings) were extracted using the LENA pro-software version (V3.4.0–143). To identify intervals with oral communication exchanges for manual transcription and coding, firstly, each recording was divided into two halves/per day. Secondly, one 5-min audio recording excerpt from the first half of the day that matched the median value of LENA-calculated CVC for the first half of the day for that child and one 5-min audio recording excerpt from the second half of the day that matched the median value of LENA-calculated CVC for the second half of the day for that child were extracted. These 5-min segments (representing times where there was a typical quantity of CVC) were used for subsequent analyses.

### Analysis strategy

#### TNW and MLU

Each child's twenty-minute audio recording was transcribed manually using a standard word processor. Specifically, for the TNW and MLU, only the initial 50 utterances were taken into consideration, adhering to the guidelines outlined in the ‘SUGAR’ analysis protocols (Sampling Utterances and Grammatical Analysis Revised; Pavelko and Owens 2019; Pavelko et al. 2020). As per ‘SUGAR’s’ definition, an utterance constitutes a sentence (e.g. I have seen a dog) or a shorter expression, separated by pauses (e.g. I like … umm … chocolate), drop in voice (e.g. I can't … *whispering* … see you), inhalations (e.g. I want to go … *deep breath* … outside), or a combination (e.g. The bus is coming and fast) (see SUGAR protocol for consideration of utterance in Appendix 1).

For TNW, all words were separated and calculated following the criteria in SUGAR. For MLU in morphemes, a set of bound morphemes was required. For instance, the breakdown resulted in two morphemes for ‘careful,’ separated as ‘care ful,’ ‘bunnies’ was dissected into ‘bunnie s,’ and ‘can’t’ was expanded as ‘cannot’. Thus, the sentence ‘she wants to sit on my seat’ was coded as comprising eight morphemes. Additional guidelines for counting morphemes can be found in Appendix 2. SUGAR is a reliable tool (Pavelko and Owens 2019; Pavelko et al. 2020) that has been used in many studies for language analysis (Pavelko et al. 2016; Owens and Pavelko 2017).

The reliability of TNW and MLU calculations was checked by comparing calculations between manual measurements undertaken independently by two of the researchers (Busch et al. 2018). Pearson product–moment correlation coefficients compared the two independent manual calculations. Results showed high consistency (TNW/50 utterances: *r* = .94; MLU/50 utterances: *r* = .93, all *p values* < .001), supporting the reliability of the manual calculation estimates.

#### Caregiver response types

A transcription sheet was created in Excel (Office 365) for coding of 20-minute audio recordings from each caregiver’s responses. The sheet encompasses four response types (WH-Q, EX, RS, and RC-Q), consistent with the classifications outlined in earlier studies (Eyberg et al. 2005; DesJardin and Eisenberg 2007; Cruz et al. 2013). Descriptions with examples of these four response types are shown in Table 3.

To calculate a frequency score for caregiver response types, the pre-identified codes for each response type (Table 4) were assigned by the first author to each adult utterance/sentence or phrase for the four transcripts for each child (2/day, for 2 days). Coding reliability was undertaken independently by two of the researchers. One coder was the first author, and the other coder was a native English-speaking researcher/expert in manual transcription, trained by a certified speech-language therapist. The first author and the second coder independently examined the recorded segments and classified the interactions for a randomly selected subset of transcripts (20% of transcripts, 200 min of recordings approximately) to verify inter-rater reliability. In the few cases of disagreement regarding the transcription, the two coders reviewed the recordings and transcripts together to reach a consensus.

**Table 4.** Descriptive data for child vocalisation count/hour, child total number of words/50 utterances, child mean length of utterance/50 utterances, and caregiver response types for children with hearing loss (CwHL) and typically developing peers with normal hearing (PwNH).

Variables	CwHL (*N* = 14)	PwNH (*N* = 20)
Quantity of Language Outcome	M (SD), Range	M (SD), Range
Child vocalisation count/ hour	5261.57 (2640.46), 1188–10068	5958.35 (2380.11), 571–10670
Child total number of word/50 utterances	102.14 (21.83), 69–134	195.70 (47.60), 124.41–266
Child mean length of utterance/50 utterances	2.14 (0.48), 1.58–2.90	4.14 (0.98), 2.54–5.64
**Caregiver response type/20 min**		
‘Wh’ Question (WH-Q)	8.20 (3.96), 2.58–14.81	16.25 (5.03), 9.82–29.68
Expansion (EX)	1.96 (1.75), 00–5.63	5.94 (5.98), 00–20.20
Reason (RS)	2.73 (3.15), 00–12.13	6.64 (5.53), 1.22–17.89
Recast Question (RC-Q)	0.49 (0.80), 00–2.60	0.97 (2.12), 00–9.63

Notes: Caregiver response types/20 min = all caregiver response types were calculated within a 20-minute timeframe.

Inter-rater reliability of coding for the four caregiver response types was determined using ICCs (intraclass correlation coefficients). ICCs are determined based on the ‘Model’ (1-way random effects, 2-way random effects, or 2-way fixed effects), the ‘Type’ (single rater/measurement or the mean of *k* raters/measurements), and the ‘Definition’ of relationship (consistency or absolute agreement) (McGraw and Wong 1996). A two-way mixed effect model was used in the current analysis. Raters were assigned a similar number of response types for scoring to obtain absolute agreement. ICCs and 95% confidence intervals were calculated using the SPSS statistical package (version 29 SPSS Inc, Chicago, IL). Inter-rater reliability among the two raters/coders for each type of response (WH*-*Q, EX, RS and RC-Q) determined using ICCs (Koo and Li 2016) showed very similar ratings for all four types of responses and excellent absolute agreement (ICC values: WH-Q = .96, EX = .98, RS = .97, RC-Q = .94).

### Analyses to address research questions

The first question focused on whether there are differences in quantity of language outcomes (CVC, TNW, and MLU), and caregiver response types (WH*-*Q, EX, RS and RC-Q) between CwHL and PwNH. Shapiro–Wilk tests showed that the data for CVC/hr and TNW/50 utterances and MLU/50 utterances were normally distributed (*p* ≥ .05) and appropriate for conducting *t*-tests (i.e. Skew ≤ |2.0|, and Kurtosis ≤ |9.0|). Thus, group comparisons for CVC, TNW and MLU were made using independent sample *t*-tests. Group comparisons for non-normally distributed data (caregiver response types; WH*-*Q, EX, RS and RC-Q) were made using Mann–Whitney *U* tests. Effect sizes for between group comparisons were obtained using Cohen’s *d* and *r* (*z*/√*N*) for parametric and non-parametric tests, respectively (Cohen 1992; Brydges 2019). The family-wise Type 1 error rate across seven measures (CVC, TNW, MLU, WH*-*Q, EX, RS and RC-Q) at the .05 level was controlled using the Holm’s sequential Bonferroni procedure (Holm 1979).

The second question focused on whether there are associations between quantity of language outcomes (CVC, TNW, and MLU), and caregiver response types (WH*-*Q, EX, RS and RC-Q), examined separately for CwHL and PwNH, using Spearman’s correlations. A multiple linear regression analysis was also undertaken with the two groups (CwHL, PwNH) combined (*N* = 34) due to the small sample size in each group (14 CwHL, 20 PwNH). All assumptions were fulfilled before running the regression model. Maternal level of education (MLE) and SES were incorporated as control variables in the multiple regression model to control for their potential impact on caregiver response types. The significance of the regression model statistics, *F *= 118.05, *p* = <.001, *R*² = 93% for TNW/50 utterances and *F* = 149.02, *p* = <.001, *R*² = 94% for MLU/50 utterances, show the overall goodness of fit of the models.

## Results

### Group differences in language outcomes and caregiver response types

Group comparisons are summarised in Table 5. Addressing research question one, independent samples *t*-tests showed CVC/hr did not differ significantly between CwHL and PwNH. However, TNW/50 utterances and MLU/50 utterances were significantly lower in CwHL than in their typically developing PwNH. Cohen’s d indicated very large effect sizes for both NTW and MLU. Mann–Whitney *U* tests showed that CwHL were exposed to significantly fewer WH*-*Q, EX, RS than their typically developing PwNH, with large effect sizes for the WH-Q and RS response types.

**Table 5.** Comparison of language outcomes (CVC, TNW, and MLU), and frequencies of caregiver response types between children with hearing loss (CwHL = 14) and typically developing peers with normal hearing (PwNH = 20).

Language outcomes			Independent samples *t*-tests		
Quantity	Group	*t*	*Df*	*p* value	Effect size
CVC/hr	CwHL	−.80	32	.214	.28
	PwNH				
TNW/50 utterances	CwHL	−6.84	32	<.001*	2.38
	PwNH				
MLU/50 utterances	CwHL	−6.95	32	<.001*	2.42
	PwNH				
Caregiver response types/20 min			Mann–Whitney U Tests		
		Mdn (IQR)	z-score	*p* value	Effect size
‘Wh’ Question (Wh-Q)	CwHL	7.03 (7.62)	−3.81	<.001*	.90
	PwNH	13.11 (5.82)			
Expansion (EX)	CwHL	1.55 (2.39)	−2.00	.045*	.45
	PwNH	2.87 (5.86)			
Reason (RS)	CwHL	2.50 (3.76)	−2.24	.025*	.86
Recast question (RC-Q)	PwNH	2.86 (5.45)	−.65	.513	.29
	CwHL	.00 (1.09)			
	PwNH	.00 (.82)			

Notes: CVC = Child vocalisation count, MLU = Child mean length of utterance, TNW = Child total number of word, Mdn = Median, IQR = Inter-quartile range, caregiver response types/20 min = all caregiver response types were calculated within a 20-minute timeframe.

*p < .05.

### Associations between language outcomes and caregiver response types

Addressing the second research question, Spearman correlation coefficients showed no significant associations between CVC and any response types for either group (Table 6).

**Table 6.** Spearman’s correlation coefficients between quantity of language outcomes and frequencies of caregiver response types in children with hearing loss (CwHL) and typically developing peers with normal hearing (PwNH).

Caregiver Response Type	Quantity of Language Outcomes	CwHL		PwNH	
		Correlation coefficient	*p* value	Correlation coefficient	*p* value
‘Wh’ question (WH-Q)	CVC/hr	.112	.703	−.165	.486
	TNW/50 utterances	.916	<.001*	.993	<.001*
	MLU/50 utterances	.999	<.001*	1.00	<.001*
Expansion (EX)	CVC/hr	.143	.625	−.135	.570
	TNW/50 utterances	.916	<.001*	.988	<.001*
	MLU/50 utterances	.997	<.001*	.996	<.001*
Reason (RS)	CVC/hr	.118	.688	.993	.508
	TNW/50 utterances	.925	<.001*	−.585	<.001*
	MLU/50 utterances	.990	<.001*	.999	<.001*
Recast question (RC-Q)	CVC/hr	.079	.787	−.287	.220
	TNW/50 utterances	.740	.002*	.931	** **< .001*
	MLU/50 utterances	.797	<.001*	.935	<.001*

Notes: CVC = child vocalisation count, MLU = child mean length of utterance, TNW = child total number of word.

*p < .05.

**Figure 1.**
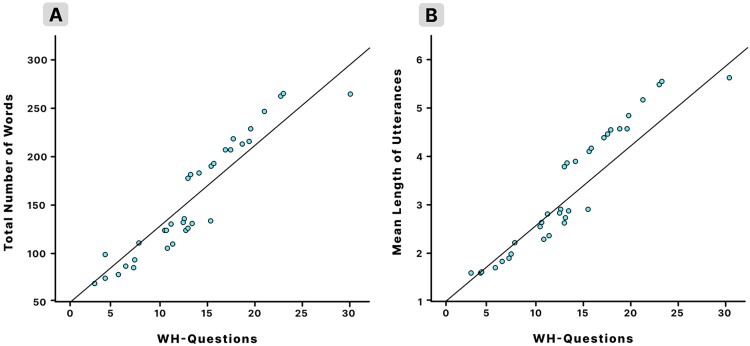
Scatter plots showing the relationship between response type (for Wh-questions) and language outcomes, **A,** Total number of words; **B,** Mean length of utterances across the two groups (*N *= 34).

However, higher TNW and MLU values were significantly associated with more WH*-*Q, EX, RS and RC-Q caregiver responses for both CwHL and PwNH. Additionally, we utilised hierarchical multiple regression analysis to examine the effect of caregiver response types (WH-Q, EX, RS, RC-Q) on language outcomes (TNW and MLU) while controlling for the impact of MLE and SES. The R-squared (R²) value revealed that 95% of the variability in TNW and MLU could be attributed to caregiver response types, considering MLE and SES. Even after excluding MLE and SES from the model, a substantial amount of variance (61%) in language outcomes was accounted for (Table 7). The regression model indicated that WH-Q and RC-Q made statistically significant contributions to both TNW and MLU (illustrated in Figure 1 for WH-Q), even with adjustments for the influence of MLE and SES. This highlights the enduring and impactful role of specific caregiver response types (WH-Q and RC-Q) in shaping language outcomes.

**Table 7.** Hierarchical multiple regression analysis for caregiver response types (Wh-Q, EX, RS, RC-Q) and language outcomes (TNW, MLU) in children.

Hierarchical Multiple Regression Analysis (A)							Hierarchical Multiple Regression Analysis (B)					
Caregiver Response Types and TNW							Caregiver Response Types and MLU					
	*Model 1*			*Model 2*			*Model 1*			*Model 2*		
Variables	*B*	*β*	SE	*B*	*β*	SE	*B*	*β*	SE	*B*	*β*	SE
Constant	51.57		63.57	3.86		19.71	1.24		1.35	.19		.38
MLE	4.32	.14	6.29	2.48	.08	1.99	.08	.11	.13	.03	.05	.04
SLD	16.42	.67	4.92	2.60	.11	1.81	.34	.66	.10	.04	.08	.04
Wh-Q				9.57	.96*	1.08				.22	1.02*	.02
EX				4.43	.37	2.48				.08	.31	.05
RS				−1.94	−.16	2.38				−.04	−.16	.05
RC-Q				−12.39	−.35*	2.42				−.25	−.34*	.05
*R*^2^	.34			.95			.34			.95		
Δ*R*^2^	.34			.61*			.34			.61*		

Notes: *N* = 34, we examined the impact of caregiver response types (Wh-Q; ‘wh’-question, EX; expansion, RS; reason, RC-Q; recast question) on language outcomes (TNW; child total number of words, MLU; child mean length of utterance). Under hierarchical multiple regression analysis (A), in model 1, we entered the control variables MLE (maternal level of education) and SES (socioeconomic status) to predict TNW, and in model 2, we entered all caregiver response types as predictors. Similarly, under hierarchical multiple regression analysis (B), in model 1, we entered the control variables MLE (maternal level of education) and SES (socioeconomic status) to predict MLU, and in model 2, we entered all caregiver response types as predictors.

*p < .05.

## Discussion

There is ample evidence in the child development literature for the impact of quantity of language input (e.g. number of adult words) on language development in young children (Hart and Risley 1995; Gilkerson and Richards 2009; Huttenlocher et al. 2010). The quality of adult language input is also important. Higher-level response types, such as expansion, recast, and open-ended questions, are associated with expressive and receptive language growth in children who are typically developing (Girolametto et al. 1999; McNeill and Fowler 1999; Rowe 2012). Both the quantity and quality of caregiver responses have also been found to be impactful for language outcomes among CwHL (Cruz et al. 2013; DesJardin et al. 2014; Ambrose et al. 2015).

Based on the LENA automatic calculations of CVC, the preschool-aged CwHL and PwNH in the current study demonstrated similar rates of vocalisation (averages of 5262/hr and 5958/hr, respectively). These vocalisation rates are much higher than the normative values indicated in the LENA Natural Language Study (Gilkerson and Richards 2009) which reported average CVC values of about 2150/hr for children at 36 months of age (the mean age of PwNH in the present study).

Despite similarities in CVC between the groups, the manual analysis of TNW and MLU showed that the linguistic content of the vocalisations of the PwNH was more mature. PwNH produced significantly higher TNW and had higher MLU, indicating that their vocalisations were more likely to be words rather than prelinguistic vocalisations. The TNW produced by PwNL in this sample (196 words) was similar to previous studies, including findings by Templin (1957) which indicated children at age 3 produced about 205 words in a 50-utterances sample. MLU is documented to be highly correlated with child age (Miller and Chapman 1981); PwNL in this sample would be expected to demonstrate a MLU of around 3.0. However, mean MLU of the PwNL in the present study was 4.14, which is higher than the expected value for children at an average age of 36 months. The caregivers of PwNH produced significantly more of three of the targeted response types (WH-Q, EX, and RS) than the caregivers of CwHL. These results are consistent with previous research which has found that CwHL are exposed to fewer high-level response types than their PwNH (DesJardin et al. 2014; Dirks and Rieffe 2019; Su and Roberts 2019). Additionally, it has been suggested that caregivers may adjust their input to match their child’s receptive, rather than expressive, language level (Girolametto et al. 1999). To this end, caregivers in the present study may have exhibited fewer of these higher-level responses because they perceived them as being at a level too high for their child’s current receptive language abilities.

The current study found no association between the rate of child vocalisations (CVC) and the caregiver response types that were investigated for either group. However, an association was found between response types (WH-Q, EX, RS, and RC-Q) and TNW and MLU. There are a few considerations for this finding. First, two of these response types (EX and RC-Q) can only be applied to child utterances which contain words. For example, if the child says ‘car’, the caregiver can expand the child’s utterance by saying ‘blue car’ or ‘car goes fast’ or recast by saying ‘is that a car?’. If the child’s vocalisation does not contain a word, EX and RC-Q cannot be applied by the caregiver. Since CwHL in this sample produced fewer words, parents in that group had fewer opportunities to apply those response types. Therefore, it makes sense that TNW and MLU were significantly associated with the use of these response types.

A second consideration is that the relationship between caregiver response types and child language outcomes is commonly believed to be bidirectional, meaning that caregiver responses to child language encourage the child to produce language, and child language productions similarly encourage caregivers to respond to their child (Tannock and Girolametto 1992). Therefore, caregivers may decrease their responses as language input to their child when they are not being ‘rewarded’ with child responses. This decreased language input, in turn, fails to stimulate child language development and could contribute to further delays. For that reason, caregivers of CwHL may benefit from structured coaching interventions to support their use of high-level response types with their children.

### Limitations

The present study has several limitations which should be considered when interpreting the results. First, this study has a small sample size, limiting generalisability of the findings. Future studies should investigate the relationship of caregiver response types and child language in a larger sample. Second, it should be noted that the reliability of LENA’s automated analyses have recently been questioned, with some studies indicating that the LENA may not be able to accurately parse vocalisations of the target child from those of other children in the environment (Cristia et al. 2020; Dilley and Houston 2021). However, acknowledging these limitations, when paired with manual analysis of variables of interest undertaken in the current study, the data collected from LENA is still believed to provide valuable insight into the language environment of the children in the study. It is also important to acknowledge the potential for errors introduced by human transcribers in the current study. Future research endeavours may benefit from utilising more reliable tools for transcription and coding. Third, it is not possible from this study to determine the direction of the relationship between caregiver responsiveness and child language outcomes; the relationship is likely complex and bidirectional. Future studies could focus on the experiences of the caregivers to understand why they communicate with their CwHL in the way that they do. Fourth, the limited sample size prevented us from separately examining the effects of high MLE and SES in factor analysis. It is important to note that in our study CwHL primarily belonged to families with relatively high SES, which is not typical of the broader population of CwHL in New Zealand, which is skewed towards lower SES (Digby et al. 2020). Additionally, most of our recorded caregiver-child interactions were from families who exclusively speak English and are of New Zealand European descent. Cultural backgrounds can influence how parents and children communicate. This presents an intriguing avenue for future research, particularly in exploring the dynamics within Māori and other indigenous families. For instance, Reese et al. (2008) observed that Māori mothers tended to share more stories about past events with their children compared to New Zealand European mothers and reported that this reminiscing was a unique, positive predictor of children's early academic skills. Potential differences in how indigenous and other cultures interact with their children using narrations, questions, and explanations, etc., is an important area for future research.

Finally, the present study did not include investigation of the impact of sign language use on caregiver responsiveness or child language outcomes. Very few studies to date have explored this; although a recent, small study found that hearing, signing mothers of CwHL produced far more initiative than responsive utterances, similar to previous findings of hearing mothers using spoken language input only (Brock 2023). Recent studies have found positive effects of sign language exposure on spoken language outcomes for CwHL (Pontecorvo et al. 2023). This is an area where more research is needed, especially in Aotearoa New Zealand where families can now access sign language learning through First Signs (https://firstsigns.co.nz/).

## Clinical implications

Many factors impact language development in CwHL, including age of identification, degree of hearing loss, age at enrolment in intervention, caregiver-child hearing status mismatch, and MLE (Ching et al. 2010; Freel et al. 2011; Vohr et al. 2011; Tomblin et al. 2015; Yoshinaga-Itano et al. 2017). One factor that has also been identified as impactful for language development in CwHL is caregiver responsiveness. Results of this preliminary investigation indicate that CwHL produced fewer words and exhibited lower MLU compared to PwNH. Further, CwHL were exposed to fewer high-level response types which are known to stimulate language development. The analysis revealed a clear association between caregiver high-level response types and child language development. These findings are consistent with findings of previous studies that indicate that CwHL (as well as language-delayed children with typical hearing) are exposed to less responsive language than children with typical language and that caregiver responsiveness is associated with child language outcomes (Girolametto et al. 1999; DesJardin et al. 2014; Levickis et al. 2014; Dirks and Rieffe 2019; Su and Roberts 2019). It should be noted that the relationship of caregiver-child interaction styles may well be bidirectional (e.g. Tannock and Girolametto 1992), meaning that caregivers may adapt their language to the level of the child rather than vice versa.

The utility of caregiver coaching in early intervention is well-established in a range of populations including children with language delays without hearing loss (Roberts et al. 2019; Heidlage et al. 2020) but is notably under-researched in dyads including CwHL (Ganek and Cardy 2021). The results of this investigation add to the evidence base indicating that caregivers of CwHL utilise fewer high-level responsive language techniques and point to the need for further research to explore the efficacy of explicit instruction and coaching on the use of these response types for caregivers of CwHL.

Family-centred programmes such as ‘It Takes Two to Talk®,’ the Hanen Program® for parents (Girolametto and Weitzman 2006), and Talking Matters (Quigan et al. 2021) are widely endorsed, however, there is a lack of evidence regarding their implementation and effectiveness in Aotearoa New Zealand. These programmes have typically been designed to focus on communication within natural everyday environments (Wood 2008; Yell et al. 2006), but they often exclude children with permanent hearing loss. Instead, such children are usually directed to specialised centres for auditory verbal/listening and spoken language therapy (Fairgray et al. 2010). Our findings suggest that family-centred coaching programmes designed for caregivers of CWHL would be a valuable additional form of support.

## Appendices

### Appendix 1

#### Instructions for Sampling Utterances and Grammatical Analysis Revised (SUGAR), (Pavelko and Owens 2019; Pavelko et al. 2020)

**Transcribe the sample directly onto computer. Only type the child’s utterances, NOT the adult. Do NOT include identifying data. Set ‘Numbering’, found on the tool bar in the ‘Paragraph’ section**, to ensure to only type 50 utterances. Remember that an utterance is a sentence or less, separated by a pause, drop in voice, inhalation or combination of these. Do not belabour the process of utterance determination. Stop when have 50 utterances.

**PROCEDURES FOR TRANSCRIBING** (Think *speed*!)

- Manual transcription using a standard Microsoft word processor. Omit immediate child imitations of the other speaker.
- Highlight all utterances that are imperative or elliptical so when we analyse each, we will know that some information has been omitted by the child.
- Omit punctuation to save time.
- Do NOT embellish the child’s utterance. In other words, don’t add morphemes that are missing.
- Type words in full even when pronunciation omits portions as follows:
  o *Talkin’* should be transcribed as ‘Talking’
  o *Gonna, wanna, gotta, hafta* should be transcribed as ‘going to, want to, got to, have to’
- Type contractions as is. In other words, *don’t* should be typed as ‘don’t’ and *I’m* as ‘I’m’
- Do NOT include fillers (uhhhh, ummm, like, you know)
- Do NOT include disfluences. **Only include the fullest form of what the child actually said**. Example: ‘He said … he says … he tell me secrets’ becomes ‘He tell me secrets’.
- Do NOT include repeated words unless it is for emphasis, as in ‘He went down down down in the cave’.
- Don’t spend an inordinate amount of time deciphering unintelligible utterances. If the entire utterance is unintelligible, omit it. If a word is unintelligible, type nonsense, such as ‘XX’ in place of the word. If an utterance contains two or more unintelligible words, omit the entire utterance.
- If an utterance contains more than two clauses joined with *and*, consider it a run-on sentence and divide as follows:
  *We went to the circus and I saw clowns and there were elephants and I got this sweet sticky stuff.but I didn’t like it so I gave it to my sister.*
    Becomes … 
  *We went to the circus and I saw clowns.*
  *There were elephants and I got this sweet sticky stuff but I didn’t like it so I gave it to my sister.*

An utterance has only one ‘and’ joining clauses. Do NOT do this with other conjunctions. Note in the previous example that the initial ‘and’ was omitted in the second utterance.

**Stop at 50 utterances. Save the document. Come out of your document. Hit Control-S for PC or Command-S for Apple/Mac to save.**

### Appendix 2

#### Supplement digital content 1

##### Calculation of total number of words (TNWs) following sugar protocols

For total number of words, we used ‘SUGAR language analysis’ is a standardised and reliable method (see detail below in process section). All utterance will be separated with space and counted all number of words used by the child. The number of words will be appeared at the bottom of the screen.

##### Calculation of mean length of utterance (MLU) following sugar protocols

**Words are already separated by a space. Now set off bound morphemes in the same way.** For example, ‘unhappily’ would be ‘un happi ly,’ ‘bunnies’ would be ‘bunnie s,’ and ‘can’t’ will be ‘ca n’t’. **Don’t worry about the spelling of the pieces or about leftover apostrophes … time is of the essence here**. For example, ‘I’ m un happi ly marrie d’ counts as 7 words, although we know it’s 7 morphemes were counting. **Follow the rules below for counting morphemes.**

RULES FOR MORPHEME COUNTING (in order to speed the process).

- Count as one morpheme (Do not separate with a space)
  Ritualised reduplications (*choo-choo*)
  Irregular past tense verbs (*went*)
  Diminutives (*doggie*)
  Auxiliary verbs
  Irregular plurals (*men*)
- Count as two morphemes (Separate with a space)
  Possessive nouns (noun + ‘*s* or *s’*)
  Plural nouns (noun + *s*)
  Third person singular present tense verbs (verb + *s*)
  Regular past tense verbs (verb + *ed*)
  Present progressive verbs (verb + *ing*)
- Count as one morpheme each word in Proper names
- Additional bound morphemes to offset with a space

**Table:**

Morpheme	Example	Morpheme	Example
-ing Adjective Gerund	Smiling girl I love hiking	-ed Adjective	Powdered sugar
-ly	Mostly	-ment	Entertainment
dis-	Dislike	re-	Refill
-er Comparative	Bigger	-y Adjective	Bumpy
-est Superlative	Biggest	-sion -tion	Discussion Invitation
-ful	Thoughtful	un-	Unhappy
-ish	Foolish		

- Count contractions (*do n’t, I’ d, he’ s, we’ ll, they’ ve*) as two morphemes

**Separate contracted words even when the stem violates traditional spelling**. We’re not grading for spelling, so just leave the pieces as is in order to save time. For example, *won’t* will end up as the two morphemes ‘wo’ and ‘n’t'. Although this seems odd, go with it.

**The number of morphemes will appear in the word count on the tool bar at the bottom of the screen. Record the number of morphemes. Divide the number of morphemes by 50.**
